# H-Ferritin Affects Cisplatin-Induced Cytotoxicity in Ovarian Cancer Cells through the Modulation of ROS

**DOI:** 10.1155/2019/3461251

**Published:** 2019-10-31

**Authors:** Alessandro Salatino, Ilenia Aversa, Anna Martina Battaglia, Alessandro Sacco, Anna Di Vito, Gianluca Santamaria, Roberta Chirillo, Pierangelo Veltri, Giuseppe Tradigo, Annalisa Di Cello, Roberta Venturella, Flavia Biamonte, Francesco Costanzo

**Affiliations:** ^1^Research Center of Biochemistry and Advanced Molecular Biology, Department of Experimental and Clinical Medicine, “Magna Græcia” University of Catanzaro, Campus Salvatore Venuta-Viale Europa, 88100 Catanzaro, Italy; ^2^Klinikumrechts der Isar, Department of Regenerative Medicine in Cardiovascular Disease, Ismaningerstr., 22 Munich, Germany; ^3^The Department of Medical and Surgical Sciences, University of Catanzaro, Italy; ^4^Unit of Obstetrics and Gynaecology, “Magna Graecia” University of Catanzaro, Catanzaro, Italy; ^5^Interdepartmental Center of Services (CIS), University “Magna Graecia” of Catanzaro, Campus Salvatore Venuta-Viale Europa, 88100 Catanzaro, Italy

## Abstract

Reactive oxygen species (ROS) mediates cisplatin-induced cytotoxicity in tumor cells. However, when cisplatin-induced ROS do not reach cytotoxic levels, cancer cells may develop chemoresistance. This phenomenon can be attributed to the inherited high expression of antioxidant protein network. H-Ferritin is an important member of the antioxidant system due to its ability to store iron in a nontoxic form. Altered expression of H-Ferritin has been described in ovarian cancers; however, its functional role in cisplatin-based chemoresistance of this cancer type has never been explored. Here, we investigated whether the modulation of H-Ferritin might affect cisplatin-induced cytotoxicity in ovarian cancer cells. First, we characterized OVCAR3 and OVCAR8 cells for their relative ROS and H-Ferritin baseline amounts. OVCAR3 exhibited lower ROS levels compared to OVCAR8 and greater expression of H-Ferritin. In addition, OVCAR3 showed pronounced growth potential and survival accompanied by the strong activation of pERK/pAKT and overexpression of c-Myc and cyclin E1. When exposed to different concentrations of cisplatin, OVCAR3 were less sensitive than OVCAR8. At the lowest concentration of cisplatin (6 *μ*M), OVCAR8 underwent a consistent apoptosis along with a downregulation of H-Ferritin and a consistent increase of ROS levels; on the other hand, OVCAR3 cells were totally unresponsive, H-Ferritin was almost unaffected, and ROS amounts met a slight increase. Thus, we assessed whether the modulation of H-Ferritin levels was able to affect the cisplatin-mediated cytotoxicity in both the cell lines. H-Ferritin knockdown strengthened cisplatin-mediated ROS increase and significantly restored sensitivity to 6 *μ*M cisplatin in resistant OVCAR3 cells. Conversely, forced overexpression of H-Ferritin significantly suppressed the cisplatin-mediated elevation of intracellular ROS subsequently leading to a reduced responsiveness in OVCAR8 cells. Overall, our findings suggest that H-Ferritin might be a key protein in cisplatin-based chemoresistance and that its inhibition may represent a potential approach for enhancing cisplatin sensitivity of resistant ovarian cancer cells.

## 1. Introduction

The oxygen-containing reactive species (ROS) are unstable by-products of cellular metabolism that are essential for several biological processes including mitochondrial and plasma membrane functioning, cell signalling and immune response [1]. The rate and magnitude of ROS production are tightly controlled by an antioxidant defense system (catalases, superoxide dismutase (SOD), glutathione peroxidase (GPx), thioredoxin (Trx), and ferritin heavy subunit (FHC)) that eliminates them over time [2, 3]. The imbalance in the circuitries of ROS production and removal leads to impairment of cell signalling, oxidative damage of cell components, and cytotoxicity [1–4]. On the other hand, recent evidences indicate that ROS are characterized by a dualistic property in determining cell fate [4, 5]. A persistent ROS overproduction may induce cellular adaptation as it occurs in many diseases, especially in cancer. Otherwise, an excessive ROS production may give rise to fatal lesions that cause cell deaths [4, 5]. Thus, many of the current chemotherapy strategies are aimed at raising ROS over the cytotoxic threshold levels in malignant cells [6–10]. To keep ROS in the prooncogenic zone, cancer cells are provided by an extensive supply of antioxidant molecules that reduce the efficacy of prooxidant drugs and enable tumor cells to acquire chemoresistance [11, 12].

Cisplatin is a prooxidant chemotherapeutic agent widely used for the treatment of ovarian cancer [13–16]. Nevertheless, ovarian cancer cells often develop cisplatin resistance by increasing the expression of the antioxidant systems [14–17]. Consequently, the final concentration of ROS evoked by cisplatin exposure is crucial for the effectiveness of this prooxidant cancer therapy [17]. For this reason, there is a strong need to develop new therapeutic strategies able to overcome platinum resistance in recurrent and metastatic ovarian cancer.

In the antioxidant enzyme family, the heavy subunit of human ferritin (H-Ferritin, FHC) acts by sequestering iron in a bioavailable and catalytically inactive form thus preventing its accumulation in the intracellular labile pool (LIP) and its participation in ROS-generating Fenton reactions [18]. The role of FHC as an antioxidant protein is underscored by the variety of mechanisms leading to its transcriptional and post-transcriptional upregulation in response to oxidative stimuli [19]. FHC expression is usually altered in cancer cells as reported in lung [20], breast [21, 22], melanoma [23], and ovarian cancer cells [24]. However, the possible relationship between FHC amounts and the aptitude of cancer cells to develop chemoresistance to cisplatin is still poorly characterized.

In the current study, we investigated whether and to which extent the modulation of H-Ferritin amounts might affect the cisplatin sensitivity in OVCAR3, a well-established *in vitro* experimental model of chemoresistant ovarian cancer cells [25], in comparison to OVCAR8 cells. Through a series of assays including FHC knockdown and forced overexpression, we demonstrate for the first time that ferritin heavy subunit, through its ability to modulate ROS amounts, is a key element in determining the response of ovarian cancer cells to cisplatin exposure.

## 2. Materials and Methods

### 2.1. Cell Lines and Cell Culture

We selected as *in vitro* experimental models two human ovarian cancer lines OVCAR3 and OVCAR8 representative of epithelial ovarian adenocarcinoma. Cells obtained from the American Type Culture Collection (ATCC, Rockville, MD, USA). According to ATCC, OVCAR3 cells derive from an ascitic metastatic site and are an appropriate model system to study cisplatin resistance in ovarian cancer. Both the cell lines are characterized by mutations in p53 gene. OVCAR3 and OVCAR8 cells were maintained in RPMI 1640 media (Sigma-Aldrich, St. Louis, MO, USA). Both culture media were supplemented with 10% fetal bovine serum at 37°C in a humidified atmosphere containing 5% CO_2_. Each cell line has been examined for mycoplasma contamination through LookOut® Mycoplasma PCR Detection Kit (Sigma-Aldrich, St. Louis, Missouri, USA).

### 2.2. Patients and Specimens

We selected a group of 28 patients with High-Grade Serous Ovarian Cancer (HGSC) who were treated at the Unit of Gynaecologic Oncology, Magna Graecia University, Germaneto, and Pugliese-Ciaccio Hospital, Catanzaro, Italy, between April 2013 and March 2016. Tissue and serum samples of patients were retrieved from our biobank to perform analysis of FHC mRNA expression. Inclusion criteria were as follows: availability of clinical data and biological samples; stage II-III-IV HGSC surgically staged. Patients with previous or concurrent cancer located in other sites, known genetic susceptibility to gynecologic or nongynecologic cancers (BRCA1-2 carriers, associated polyposis conditions (APC), Fanconi syndrome) [14], or positive family anamnesis for ovarian and/or breast cancer were excluded. Patients' clinical data are reported in Table 1.

Procedures carried out in this study were in accordance with the guidelines of the Helsinki Declaration on human experimentation and good clinical practice (GCP). Approval by the “Pugliese-Ciaccio” institutional review board (IRB number: AOPC12404) was obtained before starting patient's enrolment. Furthermore, an informed consent was obtained from all patients before processing their data from the time of hospitalization, even if data did not include any personal identifying information. Biological samples consist in surgical tissue specimens fixed in 4% paraformaldehyde and subsequently embedded in paraffin.

### 2.3. Reagents

Cisplatin was obtained from the outpatient pharmacy at Unit of Gynaecologic Oncology, Magna Graecia University, Germaneto. OVCAR3 and OVCAR8 cells were seeded in a 24-well plate in antibiotic-free medium. Cisplatin was added into the medium at various concentrations (6 *μ*M, 12 *μ*M, 24 *μ*M and 48 *μ*M). Treatments were performed at least three times on independent biological replicates. EC_50_ was calculated by using GraphPad Prism® version 5.01. N-Acetyl cysteine (NAC) was purchased from Sigma-Aldrich (Sigma-Aldrich, St. Louis, MO, USA) and used at 10 mM for 2 h.

### 2.4. ROS Detection

Intracellular ROS amounts were detected using three different methods. CellROX® Green Reagent (Thermo Fisher Scientific, Waltham, Massachusetts, USA) detects total ROS intracellular content while MitoSOX^™^ Red Indicator (Thermo Fisher Scientific, Waltham, Massachusetts, USA) specifically probes superoxide radicals. Detection was performed by immunofluorescence analysis. For immunofluorescence analysis, OVCAR3 and OVCAR8 cells were cultured on a cover slip, and upon 24 h, cells were incubated with CellROX® Green Reagent for 30 min. Both cell lines were incubated with MitoSOX^™^ Red Indicator for 10 min at 37°C. Cells were then gently washed. Cover slips were mounted on microscope slides using a mounting solution ProLong Gold antifade reagent (Thermo Fisher Scientific, Waltham, Massachusetts, USA). Images were collected using a Leica DM-IRB/TC-SP2 confocal microscopy system (63x). ROS were also determined by incubating cells with the redox-sensitive probe 2′-7′-DCF (CM-H2CFDA; Molecular Probes, Eugene, OR, USA). Analysis was performed as described in Aversa et al. [26]. Fluorescence was revealed using the Victor3 Multilabel Counter (PerkinElmer, Turku, Finland) at 485 nm and 535 nm for excitation and emission, respectively. Results were normalized on protein concentration.

### 2.5. MTT Assay

For the MTT assays, 3-[4,5-dimethylthiaoly]-2,5-diphenyltetrazolium bromide (MTT) (Sigma-Aldrich, St. Louis, MO, USA) was used. Briefly, OVCAR3 and OVCAR8 cells (50 × 10^3^ cells/well) were seeded into a 24-well plate. Upon specific treatments, fresh MTT 2 mg/mL (Sigma-Aldrich, St. Louis, MO, USA), resuspended in PBS, was added to each well containing both the cell lines; fresh MTT 2 mg/mL (Sigma-Aldrich, St. Louis, MO, USA). After 2 h incubation, culture medium was discarded and replaced with 200 *μ*L of isopropanol. Optical density was measured at 595 nm in a spectrophotometer. Analysis of OVCAR3 and OVCAR8 cell growth was performed at 0 h, 12 h, 24 h, 48 h and 72 h. For each sample, MTT assay was performed in triplicate.

### 2.6. Cell Cycle Analysis

A total of 2 × 10^5^ cells were fixed with 100% ethanol and stored at 4°C overnight. Cells were rehydrated with PBS for 10 min at RT, and then cells were stained with propidium iodide (PI) staining solution containing 50 *μ*g/mL PI (Sigma-Aldrich, St. Louis, MO, USA), 100 *μ*g/mL DNase-free RNase A (Calbiochem, La Jolla, CA), and 0.01 % NP-40 (USB, Cleveland, OH) in PBS for 60 min at room temperature. Stained cells were analyzed for cell cycle analysis in BD LSRFortessa™ X-20 (BD Biosciences, San Jose, CA) and FlowJo software.

### 2.7. Apoptosis Analysis

Apoptosis analysis was performed through the Alexa Fluor®488 Annexin V/Dead Cell Apoptosis Kit (Thermo Fisher Scientific, Waltham, Massachusetts, USA) according to the manufacturer's instructions. After staining, cells were incubated at room temperature for 15 min in the dark. Each tube was diluted with 400 *μ*L of Annexin Binding Buffer, and then, cells were analyzed by flow cytometry using the BD LSRFortessa™ X-20 (BD Biosciences, San Jose, CA) and FACSDiva7.0 program (BD Biosciences, San Jose, CA).

### 2.8. Western Blotting

Total cell lysates were prepared using RIPA buffer, as described by Aversa et al. [27]. Each protein sample (40–60 *μ*g) was separated by 10–15% SDS–PAGE and then transferred to nitrocellulose membranes. Membranes were incubated with primary antibodies at 4°C overnight. Primary antibodies against FHC (1 : 200, sc-376594), SOD1 (1 : 500, G-11, sc-17767), GPx 1/2 (1 : 500, B-6, sc-133160), c-Myc (1 : 500, C33, sc-42), CCNE1 (1 : 500, E-4, sc-377100) were purchased from Santa Cruz Biotechnology (Santa Cruz Biotechnology, Dallas, Texas). Primary antibodies against caspase 3 (1 : 1000, #9662S), AKT/pAKT (1 : 1000, 9772S/4058S), ERK/pERK (1 : 1000, 9772S/4058S), Phospho-Chk2 (Thr68) (1 : 1000, C13C1), and Chk2 (D9C6, 1 : 1000) were purchased from Cell Signalling Technology (Leiden, Netherlands). Membranes were then washed and incubated, for 2 h, with secondary antibodies HRP-conjugated goat anti-mouse IgG (1 : 2000, sc-2005) and HRP-conjugated goat anti-rabbit IgG (1 : 2000, sc-2357) (Santa Cruz Biotechnology, Dallas, Texas), and immunoreactive bands were visualized with the ECL western blotting detection system (Santa Cruz Biotechnology, Dallas, Texas). To ensure equal loading of proteins, we used goat polyclonal anti-*γ*-tubulin antibody (C-20) (1 : 2000, sc-7396, Santa Cruz Biotechnology). Experiments were performed three times and representative images are reported. Western blot densitometry was performed using ImageJ software.

### 2.9. FHC Transient Knockdown and Overexpression

OVCAR3 and OVCAR8 cells were plated into six-well plates at 5 × 10^5^ cells/well and starved overnight prior to transfection. FHC transient knockdown was performed by using a specific FHC siRNA (s5385, Thermo Fisher Scientific, Waltham, Massachusetts, USA) (OVCAR3^siFHC^ and OVCAR8^siFHC^). To ensure an optimal control, OVCAR3 and OVCAR8 cells were further transfected with *Silencer*™ Select Negative Control siRNA (Thermo Fisher Scientific, Waltham, Massachusetts, USA) (OVCAR3^Neg Control^ and OVCAR8^Neg Control^). FHC transient overexpression was performed by using a specific pc3FHC expression vector (OVCAR3^pc3FHC^ and OVCAR8^pc3FHC^) as previously reported in Zolea et al. [28]. Cells were further transiently transfected with an empty pc3DNA expression vector as negative control (OVCAR3^pc3DNA^ and OVCAR8^pc3DNA^). All transfections were performed three times using the Lipofectamine 2000 reagents according to the manufacturer's recommendations (Thermo Fisher Scientific, Waltham, Massachusetts, USA). After 48 h, FHC-specific overexpression and silencing were checked at protein levels through western blot.

### 2.10. RNA Isolation and Absolute qRT-PCR Analysis

Total RNA isolation and single-stranded complementary DNA (cDNA) generation were performed as previously reported in Di Sanzo et al. [29]. RNA from paraffin-embedded tissue specimens were obtained by a series of incubation with xylene and subsequent ethanol washes. Absolute qPCR analysis was also used to determine the expression of FHC mRNA in the 28 tumor tissue specimens. FHC expression analysis was performed by using SYBR Green qPCR Master Mix (Thermo Fisher Scientific, Waltham, Massachusetts, USA). Primers used to detect FHC were as follows: FW: 5′-CATCAACCGCCAGATCAAC-3′ and REV: 5′-GATGGCTTTCACCTGCTCAT-3′. Analysis was performed on QuantStudio 3 Applied Biosystems by Thermo Fisher Scientific. Starting from a sample of known template concentration, a 5-point 10-fold serial standard curve was prepared, and the concentration of all other samples was calculated by simple interpolation of each threshold cycle (Ct) into this standard curve. FHC mRNA expression data are reported as log (quantity, ng) and represent the mean of three independent technical replicates.

### 2.11. Statistical Analysis

Results are expressed as mean ± SD and analyzed using the unpaired Student's *t*-test or two-way ANOVA as indicated in the figure legends. GraphPad Prism® version 5.01 was used to calculate EC_50_; Sidak test was used to identify statistical significance in the EC_50_ values. FHC tumor tissue levels in chemoresistant *vs*. chemosensitive HGSC patients were compared using the nonparametric Kruskal–Wallis test to identify statistical differences between groups. The Kruskal-Wallis test has been chosen as a nonparametric alternative to one-way ANOVA and an extension of the Mann-Whitney *U* test to allow the comparison of more than two independent groups. *p* ≤ 0.05 was considered to be significant.

## 3. Results

### 3.1. OVCAR3 Cells Exhibit Lower Endogenous ROS and Higher Endogenous FHC Levels Than OVCAR8 Cells

**Figure 1:**
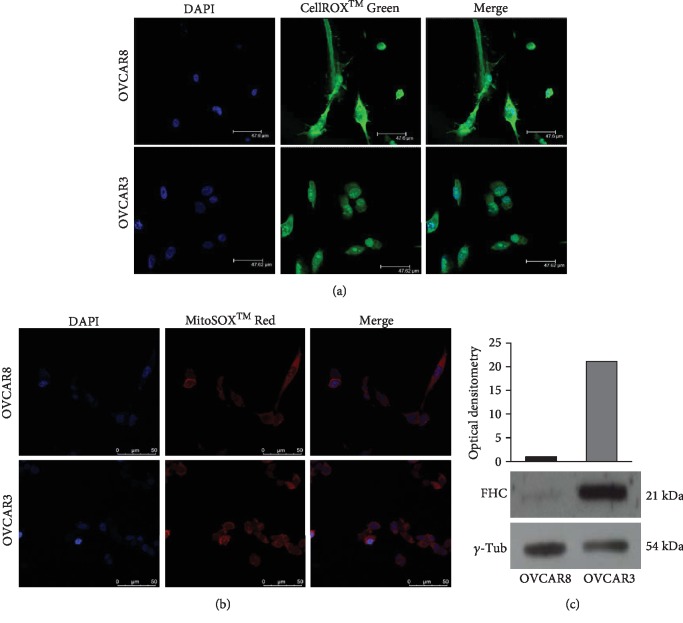
Analysis of ROS intracellular amounts and FHC antioxidant protein levels in OVCAR3 and OVCAR8 cells. (a) Immunofluorescence analysis of ROS levels in OVCAR3 and OVCAR8 cells by staining with CellROX® Green Reagent (green). Nuclei were stained with DAPI (blue). Analysis was performed in duplicate and representative images are reported. (b) Immunofluorescence analysis of superoxide radical levels in OVCAR3 and OVCAR8 cells by staining with MitoSOX™ Red Indicator (red). Nuclei were stained with DAPI (blue). Analysis was performed in duplicate and representative images are reported. (c) Representative western blot of antioxidant protein FHC in OVCAR3 and OVCAR8 cells. *γ*-Tubulin was used as internal control. WB has been quantified by using ImageJ software and optical densitometry is reported. WB analysis was performed three times and results were reproducible.

We selected as *in vitro* experimental models OVCAR3 and OVCAR8 cell lines representative of epithelial ovarian adenocarcinoma. OVCAR3 has been chosen as experimental model for studying cisplatin chemoresistance [25]. First, we performed ROS analysis in OVCAR3 and OVCAR8 cells by using two fluorogenic probes: CellROX® Green Reagent able to detect total intracellular ROS content and MitoSOX™ Red Indicator able to selectively detect superoxide radicals. As shown in Figures 1(a) and 1(b), fluorescence microscopy highlights that CellROX® Green fluorescence intensity was significantly higher in OVCAR8 cells compared to OVCAR3 cells. Conversely, MitoSOX staining showed only a slightly different intensity between the two cell lines. These results suggest that OVCAR8 cells are characterized by higher levels of total ROS content compared to OVCAR3 cells. The superoxide radical contribution to this different levels appeared inconsistent. Differences in baseline ROS amounts might reflect, in principle, diverse expression of antioxidant enzymes. Thus, we analyzed the expression of FHC protein, belonging to the antioxidant system, in both OVCAR3 and OVCAR8 cells. Representative western blot analyses and relative densitometry reported in Figure 1(c) highlight that the antioxidant protein FHC was consistently more expressed in OVCAR3 compared to OVCAR8 cells. This behaviour was mirrored by the expression of the other two antioxidant enzymes SOD1 and GPx ([Supplementary-material supplementary-material-1]).

### 3.2. OVCAR3 Cells Show Enhanced Cell Cycle S-Phase and Cell Growth

**Figure 2:**
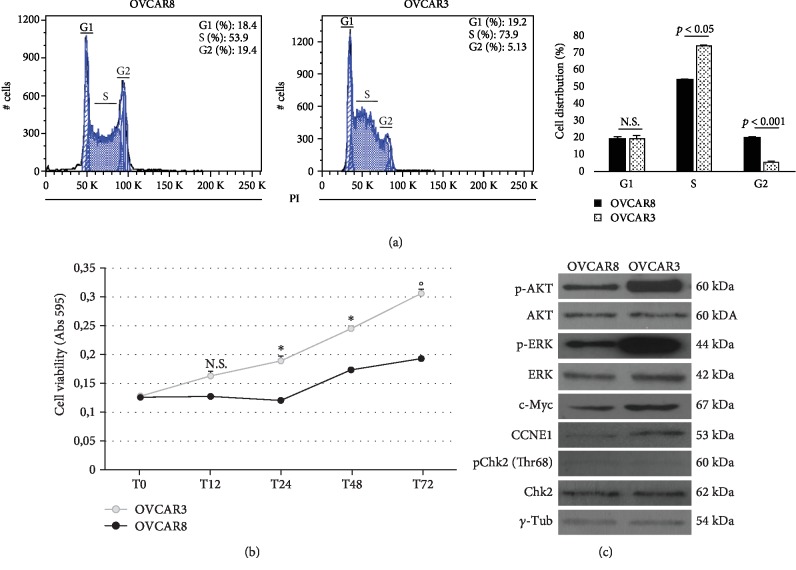
OVCAR3 cells exhibit increased growth potential compared to OVCAR8 cells. (a) Cell cycle FACS analysis of OVCAR3 and OVCAR8 cells stained with PI. The experiments were performed in triplicate. Representative plots of a single experiment (left); histograms showing the mean ± SD of three independent experiments (right). ^∗^*p* value < 0.05, OVCAR3 *vs*. OVCAR8. (b) MTT analysis of OVCAR3 and OVCAR8 cell growth at 12 h, 24 h, 48 h, and 72 h. Data are reported as absorbance measured at 595 nm and shown as mean ± SD of three independent replicates (^∗^*p* < 0.05, OVCAR3 *vs*. OVCAR8); (°*p* < 0.01, OVCAR3 *vs*. OVCAR8); N.S.: not significant. (c) Representative WB of c-Myc, cyclin E1 (CCNE1), pChk2 (Thr68), pERK1/2, and pAKT in OVCAR3 and OVCAR8 cells. *γ*-Tubulin was used as internal control. WB analysis was performed three times and results were reproducible.

We next assessed whether the differences in intracellular baseline ROS amount and FHC protein levels were paralleled by different cancer cell growth.  Figure 2(a) shows a representative plot and histograms indicating the mean ± SD of three cell cycle cytofluorimetric analyses, performed by staining cells with PI solution. Results highlight that a significant higher percentage of OVCAR3 cells were in S-phase compared to OVCAR8 cells (S%: 73.9 ± 0.3*vs*. 53.9 ± 0.7, *p* < 0.05). Accordingly, results from the MTT analysis show that OVCAR3 exhibited an enhanced cell growth potential at 24 h, 48 h, and 72 h (Figure 2(b)). As reported in Figure 2(c), OVCAR3 cells were also characterized by consistent overexpression of the specific S-phase cyclin E1 (CCNE1) along with increased expression of the proto-oncogene c-Myc and enhanced phosphorylation of ERK1/2 and AKT compared to OVCAR8 cells. On the contrary, no phosphorylation of the S-phase cyclin-dependent kinase Chk2 (Thr68) was observed in OVCAR3 and OVCAR8 cells. Optical densitometry of each WB analysis is reported in [Supplementary-material supplementary-material-1].

### 3.3. Cisplatin Treatment Induces Significant FHC Downregulation and ROS Increase Exclusively in Chemosensitive OVCAR8 Cells

**Figure 3:**
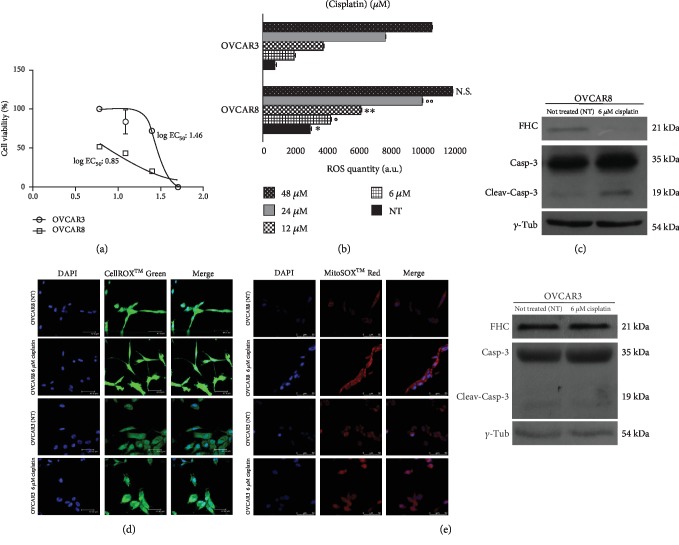
OVCAR8 cells are characterized by ROS accumulation and FHC downregulation upon 6 *μ*M cisplatin treatment. (a) Cell viability assay performed by MTT analysis in OVCAR3 and OVCAR8 cells treated with 6 *μ*M, 12 *μ*M, 24 *μ*M and 48 *μ*M of cisplatin for 24 h. Cisplatin concentrations are reported as log [cisplatin (*μ*M)]. Cell viability is expressed as percentage (%). Treatments were performed at least three times on independent biological replicates and the mean concentration of the drug that gives half-maximal response (log EC_50_) was used to compare cytotoxicity. (b) Quantification of ROS amounts through DCFDA staining in OVCAR3 and OVCAR8 untreated (NT) and upon treatment with 6 *μ*M, 12 *μ*M, 24 *μ*M and 48 *μ*M cisplatin for 24 h. Data represent the mean ± SD of three biological replicates. ^∗^*p* value < 0.01 OVCAR3 NT *vs*. OVCAR8 NT; °*p* value < 0.01 OVCAR3 6 *μ*M cisplatin *vs*. OVCAR8 6 *μ*M cisplatin; ^∗∗^*p* value < 0.05 OVCAR3 12 *μ*M cisplatin *vs*. OVCAR8 12 *μ*M cisplatin; °°*p* value < 0.05 OVCAR3 24 *μ*M cisplatin *vs*. OVCAR8 24 *μ*M cisplatin; N.S.: not significant: OVCAR3 48 *μ*M cisplatin *vs*. OVCAR8 48 *μ*M cisplatin. (c) Representative western blot of FHC, cleaved caspase 3, and caspase 3 in OVCAR3 and OVCAR8 untreated (NT) and upon treatment with 6 *μ*M cisplatin for 24h. *γ*-Tub was used as internal control. WB analysis was performed three times and results were reproducible. (d) Immunofluorescence analysis of ROS levels in untreated (NT) and treated with 6 *μ*M cisplatin OVCAR3 and OVCAR8 cells by staining with CellROX® Green Reagent (green). Nuclei were stained with DAPI (blue). Analysis was performed in duplicate and representative images are reported. (e) Immunofluorescence analysis of superoxide radical levels untreated (NT) and treated with 6 *μ*M cisplatin OVCAR3 and OVCAR8 cells by staining with MitoSOX™ Red Indicator (red). Nuclei were stained with DAPI (blue). Analysis was performed in duplicate and representative images are reported.

The sensitivity of OVCAR3 and OVCAR8 cells to cisplatin was determined by treating both cell lines with increasing concentrations of the drug (6 *μ*M, 12 *μ*M, 24 *μ*M and 48 *μ*M). After 24 h, we performed the MTT assay to monitor cell viability following treatment. As shown in Figure 3(a), we found that OVCAR3 cells were, overall, more resistant to treatment than OVCAR8 cells (log EC_50_ OVCAR3 *vs*. log EC_50_ OVCAR8: 1.46 ± 0.06 vs. 0.85 ± 0.05, *p* < 0.0001). In particular, at the lowest cisplatin concentration (6 *μ*M) OVCAR8 cell viability was almost halved while OVCAR3 cells were totally unresponsive. DCFDA luminometric analysis highlighted that the extent of ROS accumulation induced by cisplatin treatments in OVCAR8 cells was significantly higher than that induced in OVCAR3 cells at each concentration apart from 48 *μ*M (Figure 3(b)). Next, we observed that the exposure to 6 *μ*M cisplatin induced a clear cleavage of caspase 3 in OVCAR8 cells and not in OVCAR3 cells (Figure 3(c)). Furthermore, FHC protein levels showed a completely different behaviour between the two cell lines; at 6 *μ*M cisplatin, FHC was consistently downregulated in OVCAR8 cells whereas it was almost unaffected in OVCAR3 cells (Figure 3(c)). Optical densitometries of WB are reported in Fig. [Supplementary-material supplementary-material-1]. The analysis of SOD1 and GPx protein levels upon 6 *μ*M cisplatin showed, instead, a slight increase in both the cell lines ([Supplementary-material supplementary-material-1]). Furthermore, immunofluorescence analysis highlighted that 6 *μ*M cisplatin induced a significant increase in both total ROS and superoxide radical levels in the drug-responsive OVCAR8 cells compared to OVCAR3 resistant cells (Figures 3(d) and 3(e)).

### 3.4. Modulation of Intracellular FHC Levels Affects Sensitivity to Cisplatin in OVCAR3 and OVCAR8 Cells

**Figure 4:**
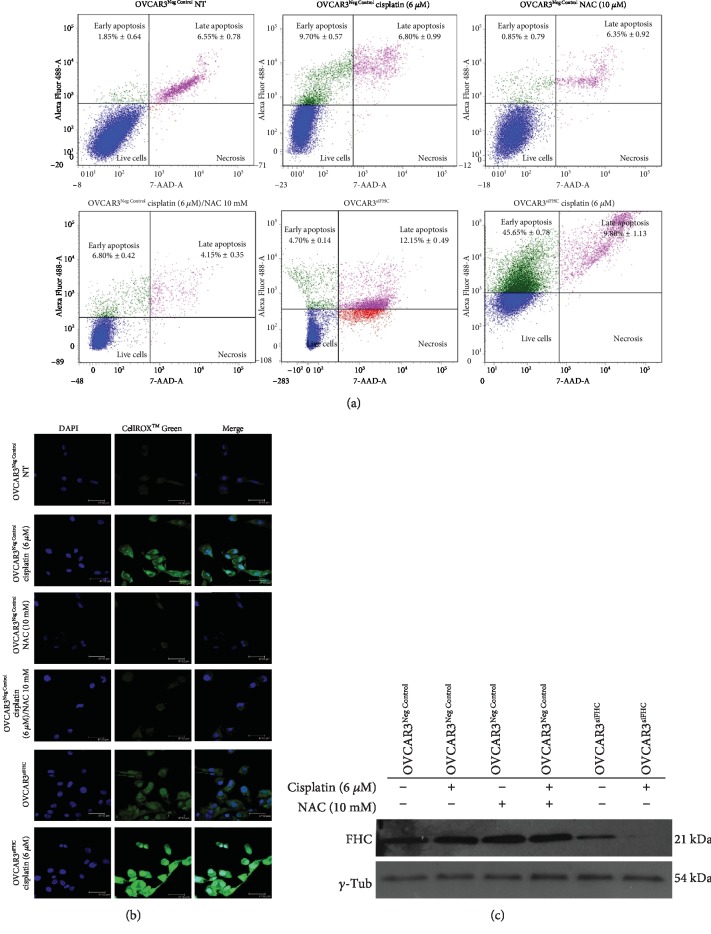
FHC knockdown improves OVCAR3 response to 6 *μ*M cisplatin by increasing ROS production. (a) Representative plots of Annexin V/7-AAD apoptosis assays in OVCAR3^Neg Control^, OVCAR3^Neg Control^ (6 *μ*M) cisplatin, OVCAR3^Neg Control^ 10 mM NAC, OVCAR3^Neg Control^ (6 *μ*M) cisplatin/10 mM NAC, OVCAR3^siFHC^, and OVCAR3^siFHC^ (6 *μ*M) cisplatin. Cisplatin treatment was performed for 24 h while NAC treatment was performed for 2 h. FACS plots are representative of single experiments. Values are expressed as mean ± SD of three biological replicates. (b) Immunofluorescence analysis of ROS levels in OVCAR3^Neg Control^, OVCAR3^Neg Control^ (6 *μ*M) cisplatin, OVCAR3^Neg Control^ 10 mM NAC OVCAR3^Neg Control^ (6 *μ*M) cisplatin/10 mM NAC, OVCAR3^siFHC^, and OVCAR3^siFHC^ (6 *μ*M) cisplatin, by staining with CellROX® Green Reagent (green). Nuclei were stained with DAPI (blue). (c) Representative western blot of FHC in OVCAR3^Neg Control^, OVCAR3^Neg Control^ (6 *μ*M) cisplatin, OVCAR3^Neg Control^ 10 mM NAC, OVCAR3^Neg Control^ (6 *μ*M) cisplatin/10 mM NAC, OVCAR3^siFHC^, and OVCAR3^siFHC^ (6 *μ*M) cisplatin. *γ*-Tubulin was used as internal control. WB analysis was performed three times and results were reproducible.

Here, we asked whether a change in FHC levels might affect the EOC cell response to cisplatin. To this, we first transiently transfected OVCAR3 cells with a specific FHC siRNA (OVCAR3^siFHC^) or negative control (OVCAR3^Neg Control^) for 48 h. Annexin V/7-AAD cytofluorimetric analysis showed that 6 *μ*M cisplatin was unable to induce a consistent apoptosis in OVCAR3^Neg Control^ (early apoptosis: 9.70% ± 0.57; late apoptosis: 6.80% ± 0.99). On the contrary, the same drug concentration promoted a significant increase of apoptotic cell death in OVCAR3^siFHC^ cells (early apoptosis: 45.65% ± 0.78; late apoptosis: 9.80% ± 1.13) compared to either untreated OVCAR3^Neg Control^ (*p* < 0.05) or OVCAR3^Neg Control^ treated with 6 *μ*M cisplatin alone (*p* < 0.05). Apoptosis assays performed in OVCAR3^Neg Control^ cells treated with (i) 10 mM N-acetyl-cysteine (NAC) alone and (ii) 10 mM N-acetyl-cysteine (NAC) in combination with 6 *μ*M cisplatin and in untreated OVCAR3^siFHC^ revealed no considerable changes. Results of three independent biological replicates are reported as mean ± SD in Table 2 as well as in Figure 4(a).

Accordingly, detection with CellROX® Green Reagent revealed that the ROS amounts evoked by 6 *μ*M cisplatin treatment in combination with FHC knockdown in OVCAR3 cells (OVCAR3^siFHC^ cisplatin 6 *μ*M) were consistently higher than those induced by either cisplatin treatment alone (OVCAR3^Neg Control^ cisplatin 6 *μ*M) or FHC silencing alone (OVCAR3^siFHC^) (Figure 4(b)). No considerable changes have been observed in OVCAR3 cells treated with 10 mM NAC (OVCAR3^Neg Control^ NAC 10 mM) in comparison with OVCAR3^Neg Control^ untreated cells. As expected, NAC treatment reduced ROS accumulation in OVCAR3 cells treated with 6 *μ*M of cisplatin (OVCAR3^Neg Control^ cisplatin 6 *μ*M/NAC 10 mM). In addition, western blot analysis revealed that 6 *μ*M of cisplatin exposure led to a further downregulation of FHC protein levels in OVCAR3^siFHC^ cells; conversely, cisplatin left FHC levels unaltered in OVCAR3^Neg Control^ cells (Figure 4(c)).

**Figure 5:**
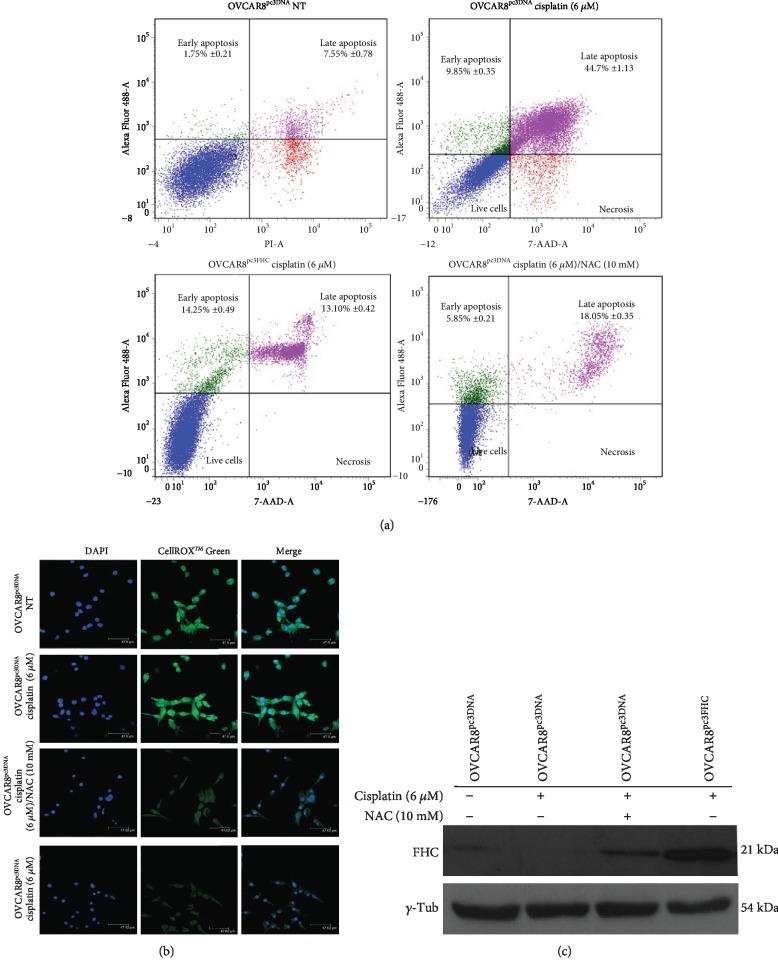
FHC overexpression or NAC treatment reduces OVCAR8 response to cisplatin. (a) Representative plots of Annexin V/7-AAD apoptosis assays in OVCAR8^pc3DNA^, OVCAR8^pc3DNA^ (6 *μ*M) cisplatin, OVCAR8^pc3DNA^ (6 *μ*M) cisplatin/10 mM NAC, and OVCAR8^pc3FHC^ (6 *μ*M) cisplatin. Cisplatin treatment was performed for 24 h while NAC treatment was performed for 2 h. FACS plots are representative of single experiments. Values are expressed as mean ± SD of three biological replicates. (b) Immunofluorescence analysis of ROS levels in OVCAR8^pc3DNA^, OVCAR8^pc3DNA^ (6 *μ*M) cisplatin, OVCAR8^pc3DNA^ (6 *μ*M) cisplatin/10 mM NAC, and OVCAR8^pc3FHC^ (6 *μ*M) cisplatin, by staining with CellROX® Green Reagent (green). Nuclei were stained with DAPI (blue). (c) Representative western blot of FHC in OVCAR8^pc3DNA^, OVCAR8^pc3DNA^ (6 *μ*M) cisplatin, OVCAR8^pc3DNA^ (6 *μ*M) cisplatin/10 mM NAC, and OVCAR8^pc3FHC^ (6 *μ*M) cisplatin. *γ*-Tubulin was used as internal control. WB analysis was performed three times and results were reproducible.

Next, we performed FHC overexpression in OVCAR8 cells. As shown in Figures 5(a) and 5(c), the forced FHC overexpression significantly protected OVCAR8 cells from 6 *μ*M cisplatin-induced cytotoxicity (OVCAR3^pc3FHC^ cisplatin (6 *μ*M) *vs*. OVCAR8^pc3DNA^ cisplatin (6 *μ*M), *p* < 0.05). Similar results were obtained when OVCAR8 cells were treated with 6 *μ*M cisplatin for 24 h in combination with 10 mM of ROS scavenger NAC for 2 h (OVCAR8^pc3DNA^ cisplatin (6 *μ*M)/NAC (10 mM) *vs*. OVCAR8^pc3DNA^ cisplatin (6 *μ*M), *p* < 0.05). Results of three independent biological replicates are reported as mean ± SD in Table 3. Accordingly, detection with CellROX® Green Reagent further revealed that FHC overexpression, as well as NAC treatment, consistently reduced ROS amounts evoked by 6 *μ*M cisplatin treatment alone in OVCAR8 cells (Figures 5(b)).

### 3.5. FHC Tissue Levels Are Higher in Chemoresistant High-Grade Serous Ovarian Cancer (HGSC) Patients Compared to Chemosensitive Ones

In light of the *in vitro* findings, we performed absolute qPCR analysis of *FHC* mRNA tissue levels in a small cohort of 28 patients with High-Grade Serous Ovarian Cancer (HSGC, stages II, III, and IV) treated with platinum-based chemotherapy, among which 13 were chemoresistant and 15 were chemosensitive. As shown in the box plot in [Supplementary-material supplementary-material-1], statistical Kruskal–Wallis test suggests that patients with resistance to chemotherapy may be characterized by higher FHC levels compared to the chemosensitive ones. However, data do not reach the statistical significance.

## 4. Discussion

Despite considerable efforts for developing novel and more efficient therapeutic strategies, ovarian cancer patients often suffer from aggressive and therapy-resistant disease characterized by poor prognosis and high mortality [14, 30, 31]. Cisplatin is a prooxidant chemotherapeutic agent largely used as first-line therapy in ovarian cancer; however, its efficacy is quite limited since most patients ultimately die with platinum-resistant disease [30, 31]. Numerous evidences indicate that altered redox balance, which is now widely considered as one of the main cancer hallmarks, can be pivotal in the resistance to antitumor agents including cisplatin [1, 2, 4].

As a consequence of genetic, metabolic, and microenvironment-related aberrations, cancer cells are subjected to persistent prooxidant stimuli that ultimately increase baseline ROS levels and promote tumor growth by inducing genomic instability and metabolism reprogramming [32]. However, tumor cells have developed an efficient ROS detoxification system through which they gain advantage when subjected to further prooxidant conditions. This dependency from the antioxidant systems represents a specific vulnerability so as the current used prooxidant chemotherapeutic agents act by increasing oxidative stress above the toxicity threshold [1–5].

In this study, by analyzing changes of intracellular ROS levels, we explored the role of FHC, an important antioxidant enzyme, in the development of resistance to cisplatin-based therapy in ovarian cancer cells.

FHC, the heavy subunit of the human ferritin, has a ferroxidase activity through which it safely stores iron in catalytically inactive Fe^3+^ form thus tightly controlling the homeostasis of the labile prooxidant iron pool [33]. We and others have previously demonstrated that FHC is a downstream effector of NFkB-mediated inhibition of the oxidative stress-induced apoptosis [26, 34]. In addition, FHC is transcriptionally upregulated by the antioxidant transcription factor Nrf2 to maintain iron and redox homeostasis [35, 36].

As an *in vitro* experimental model, we selected OVCAR3 and OVCAR8 cell lines as representative of ovarian cancer cells. In particular, OVCAR3 cells have been selected as cisplatin refractory cell line established from metastatic ascites of a patient with ovarian adenocarcinoma [25].

Overall, our results strongly suggest that the antioxidant properties of FHC play a key role in determining the response of ovarian cancer cells to cisplatin treatment. First, we observed that the chemoresistant OVCAR3 cells are characterized by higher constitutive FHC levels and by lower endogenous ROS content in comparison to OVCAR8 cells. Moreover, prooxidant cisplatin treatment affects FHC levels in OVCAR8 cells by inducing its downregulation while it leaves unchanged FHC amounts in OVCAR3 cells. These effects appear to be selective for FHC since other two antioxidant enzymes, namely SOD1 and GPx, were not consistently modified upon cisplatin exposure. Recent evidences indicate that H-Ferritin may undergo degradation in cells exposed to anticancer compound and this is accompanied by intracellular iron accumulation and increase in iron-dependent ROS production [37]. Indeed, we noticed that in OVCAR8 cells the downregulation of FHC mediated by cisplatin exposure was accompanied by an enormous increase in ROS accumulation that likely exceed the cytotoxic threshold levels. Conversely, in OVCAR3 cells the cisplatin hit was insufficient to push ROS production over the cytotoxic levels.

In the past decades, three main approaches have been proposed to exploit the cancer cell killing potential of ROS: (i) enhancing the generation of ROS in tumor cells by increasing the dose of a single prooxidant chemotherapeutic drug, (ii) combination of conventional anticancer agents with natural compounds that increase ROS production, the so-called “ROS+ROS concept,” and (iii) inhibition of the antioxidant defense system of tumor cells. Remarkably, the first two approaches are very challenging to translate from *in vitro* models to *in vivo* conditions because of significant side effects [10, 38, 39]. On the contrary, disabling key antioxidant systems in the presence of ROS inducers represents the most promising new anticancer strategy in resistant tumor cells. Indeed, impairing antioxidant capacity, such as Nrf2, SOD, and GPx, has emerged as a good strategy to target many cancer types [40]. Here, we proved, for the first time, that modulation of intracellular H-Ferritin (FHC) protein is able to condition ovarian cancer cell response to cisplatin thus adding this molecule to the targetable antioxidant protein panel. The relevance of FHC/ROS axis in modulating ovarian cancer cell response to cisplatin has been demonstrated by FHC knockdown or forced overexpression in our *in vitro* system. The knockdown of FHC, by using a specific siRNA, is accompanied by a significant augment in the cytotoxic effects of cisplatin in the drug-resistant OVCAR3 cells. Accordingly, an overexpression of FHC in the drug-sensitive OVCAR8 cells suppresses the cytototoxic effects of the drug at a level comparable to that obtained by scavenging ROS through NAC treatment. The fundamental mechanism through which FHC knockdown is able to restore OVCAR3 sensitivity to cisplatin appears to be strictly related to its antioxidant properties and to its capacity to lead the effective final amounts of ROS over those evoked by the cisplatin treatment alone.

At last, we also analyzed FHC cancer tissue levels in 28 patients with HGSC receiving a platinum-based chemotherapy. Although suggestive of a possible association between high levels of FHC and chemoresistance, the collected data do not reach the statistical significance. This trend prompted us to increase, in future studies, the analyzed cohort of patients to provide additional arguments in favor of the importance of estimating ROS amounts and FHC status to improve the therapeutic outcomes in treatment of ovarian cancer.

## 5. Conclusions

In conclusion, our data demonstrate for the first time the association of FHC/ROS axis with cisplatin resistance in ovarian cancer cells. Furthermore, we propose that inhibition of FHC might be a potential approach for restoring cisplatin sensitivity of resistant ovarian cancer cells. The conjugation of siRNA carrier system with ligands that exhibit high affinity to specific receptors overexpressed in ovarian cancer cells could make feasible this approach also *in vivo*.

## Figures and Tables

**Table 1:** Clinical, pathological, and surgical characteristics of patients with High-Grade Serous Ovarian Cancer (HGSC).

	HGSC (*n* = 28)
Age (years)	59.5 ± 19.0
FIGO stage (*n*, %)	
Stage II	7 (25.0)
Stage III	15 (53.6)
Stage IV	6 (21.4)
Primary debulking surgery (*n*, %)	17 (100)
Chemotherapy (*n*, %)	
Platinum+Taxol+Beva	28 (100)
Response to chemotherapy	
Resistant	13 (46.4)
Sensitive	15 (53.6)
Major comorbidities (*n*, %)	5 (17.8)
Follow-up (months)	26.2 ± 18.2

**Table 2:** Data analysis of Annexin/7-AAD cytofluorimetric apoptosis assays in OVCAR3 cells.

Samples	Early apoptosis (% ± SD)	Late apoptosis (% ± SD)	Live cells (% ± SD)
OVCAR3^Neg Control^ NT^∗^	1.85 ± 0.64	6.55 ± 0.78	90.45 ± 1.77
OVCAR3^Neg Control^ cisplatin (6 *μ*M)^∗∗^	9.70 ± 0.57	6.80 ± 0.99	83.85 ± 1.20
OVCAR3^Neg Control^ NAC (10 mM)	0.85 ± 0.79	6.35 ± 0.92	92.18 ± 1.77
OVCAR3^Neg Control^ cisplatin (6 *μ*M)/NAC (10 mM)	6.80 ± 0.42	4.15 ± 0.35	89.45 ± 0.92
OVCAR3^siFHC^	4.70 ± 0.14	12.15 ± 0.49	76.65 ± 1.06
OVCAR3^siFHC^ cisplatin (6 *μ*M)	45.65 ± 0.78	9.80 ± 1.13	43.85 ± 0.49

^∗^OVCAR3^siFHC^ cisplatin (6 *μ*M) vs. OVCAR3^Neg Control^ NT, *p* value < 0.05 (two-way ANOVA test). ^∗∗^OVCAR3^siFHC^ cisplatin (6 *μ*M) vs. OVCAR3^Neg Control^ cisplatin (6 *μ*M), *p* value < 0.05 (two-way ANOVA test).

**Table 3:** Data analysis of Annexin/7-AAD cytofluorimetric apoptosis assays in OVCAR8 cells.

Samples	Early apoptosis (% ± SD)	Late apoptosis (% ± SD)	Live cells (% ± SD)
OVCAR8^pc3DNA^ NT	1.75 ± 0.21	7.55 ± 0.78	82.75 ± 1.20
OVCAR8^pc3DNA^ cisplatin (6 *μ*M)	9.85 ± 0.35	44.70 ± 1.13	42.35 ± 1.20
OVCAR8^pc3DNA^ cisplatin (6 *μ*M)/NAC (10 mM)^∗^	5.85 ± 0.21	18.05 ± 0.35	71.70 ± 0.99
OVCAR3^pc3FHC^ cisplatin (6 *μ*M)^∗∗^	14.25 ± 0.49	13.10 ± 0.42	71.25 ± 1.63

^∗^OVCAR8^pc3DNA^ cisplatin (6 *μ*M)/NAC (10 mM) vs. OVCAR8^pc3DNA^ cisplatin (6 *μ*M), *p* value < 0.05 (two-way ANOVA test). ^∗∗^OVCAR3^pc3FHC^ cisplatin (6 *μ*M) vs. OVCAR8^pc3DNA^ cisplatin (6 *μ*M), *p* value < 0.05 (two-way ANOVA test).

## Data Availability

The data used to support the findings of this study are included within the article.

## Supplementary Materials

Supplementary Materials Fig.S1: WB analysis of SOD1, GPx, CCNE1, c-Myc, ERK/pERK, AKT/pAKT, and Chk2/pChk2 in OVCAR3 and OVCAR8 cells. Representative western blot of antioxidant proteins SOD1 and GPx, CCNE1, and c-Myc together with ERK/AKT/Chk2 phosphorylation in OVCAR3 and OVCAR8 cells. *γ*-Tubulin was used as internal control. Each plot has been quantified by using ImageJ software and optical densitometry is reported. WB analysis was performed three times and results were reproducible. Fig. S2: WB analysis of FHC, caspase 3/cleaved caspase 3, SOD1, and GPx in OVCAR3 and OVCAR8 cells untreated or treated with 6 *μ*M cisplatin. Representative western blot of FHC, caspase 3/cleaved caspase 3, SOD1, and GPx in OVCAR3 and OVCAR8 cells untreated (NT) or treated with 6 *μ*M cisplatin. *γ*-Tubulin was used as internal control. Each plot has been quantified by using ImageJ software and optical densitometry is reported. WB analysis was performed three times and results were reproducible. Fig. S3: WB analysis of FHC, caspase 3/cleaved caspase 3, SOD1, and GPx in OVCAR3 and OVCAR8 cells upon several treatments. Representative western blot of FHC in OVCAR3^Neg Control^, OVCAR3^Neg Control^ (6 *μ*M) cisplatin, OVCAR3^Neg Control^ 10 mM NAC, OVCAR3^Neg Control^ (6 *μ*M) cisplatin/10 mM NAC (6 *μ*M) cisplatin, OVCAR3^siFHC^, OVCAR3^siFHC^ (6 *μ*M) cisplatin, OVCAR8^pc3DNA^, OVCAR8^pc3DNA^ (6 *μ*M) cisplatin, OVCAR8^pc3DNA^ (6 *μ*M) cisplatin/10 mM NAC, and OVCAR8^pc3FHC^ (6 *μ*M) cisplatin. *γ*-Tubulin was used as internal control. Each plot has been quantified by using ImageJ software and optical densitometry is reported. WB analysis was performed three times and results were reproducible. Fig. S4: analysis of FHC tumor tissue levels in 28 HGSC patients. Representative images of ovarian cancer tissue specimens (left). Box plot depicting FHC mRNA levels, expressed as log (ng), as assessed by absolute qPCR analysis in cancer tissue specimens of chemoresistant HGSC patients (*n* = 13) and chemosensitive HGSC patients (*n* = 15) (right). Data are not statistically significant (N.S.).
