# Supplementary material for: H-Ferritin Affects Cisplatin-Induced Cytotoxicity in Ovarian Cancer Cells through the Modulation of ROS
Source: Oxid Med Cell Longev. 2019 Oct 31;2019:3461251. doi: 10.1155/2019/3461251 (PMC6875340; doi:10.1155/2019/3461251)
Supplement: Supplementary Materials — Fig.S1: WB analysis of SOD1, GPx, CCNE1, c-Myc, ERK/pERK, AKT/pAKT, and Chk2/pChk2 in OVCAR3 and OVCAR8 cells. Representative western blot of antioxidant proteins SOD1 and GPx, CCNE1, and c-Myc together with ERK/AKT/Chk2 phosphorylation in OVCAR3 and OVCAR8 cells. γ-Tubulin was used as internal control. Each plot has been quantified by using ImageJ software and optical densitometry is reported. WB analysis was performed three times and results were reproducible. Fig. S2: WB analysis of FHC, caspase 3/cleaved caspase 3, SOD1, and GPx in OVCAR3 and OVCAR8 cells untreated or treated with 6 μM cisplatin. Representative western blot of FHC, caspase 3/cleaved caspase 3, SOD1, and GPx in OVCAR3 and OVCAR8 cells untreated (NT) or treated with 6 μM cisplatin. γ-Tubulin was used as internal control. Each plot has been quantified by using ImageJ software and optical densitometry is reported. WB analysis was performed three times and results were reproducible. Fig. S3: WB analysis of FHC, caspase 3/cleaved caspase 3, SOD1, and GPx in OVCAR3 and OVCAR8 cells upon several treatments. Representative western blot of FHC in OVCAR3Neg Control, OVCAR3Neg Control (6 μM) cisplatin, OVCAR3Neg Control 10 mM NAC, OVCAR3Neg Control (6 μM) cisplatin/10 mM NAC (6 μM) cisplatin, OVCAR3siFHC, OVCAR3siFHC (6 μM) cisplatin, OVCAR8pc3DNA, OVCAR8pc3DNA (6 μM) cisplatin, OVCAR8pc3DNA (6 μM) cisplatin/10 mM NAC, and OVCAR8pc3FHC (6 μM) cisplatin. γ-Tubulin was used as internal control. Each plot has been quantified by using ImageJ software and optical densitometry is reported. WB analysis was performed three times and results were reproducible. Fig. S4: analysis of FHC tumor tissue levels in 28 HGSC patients. Representative images of ovarian cancer tissue specimens (left). Box plot depicting FHC mRNA levels, expressed as log (ng), as assessed by absolute qPCR analysis in cancer tissue specimens of chemoresistant HGSC patients (n = 13) and chemosensitive HGSC patients (n = 15) (right). Data are not st [file 3461251.f1.pdf]

Supplementary Materials

Figure S1

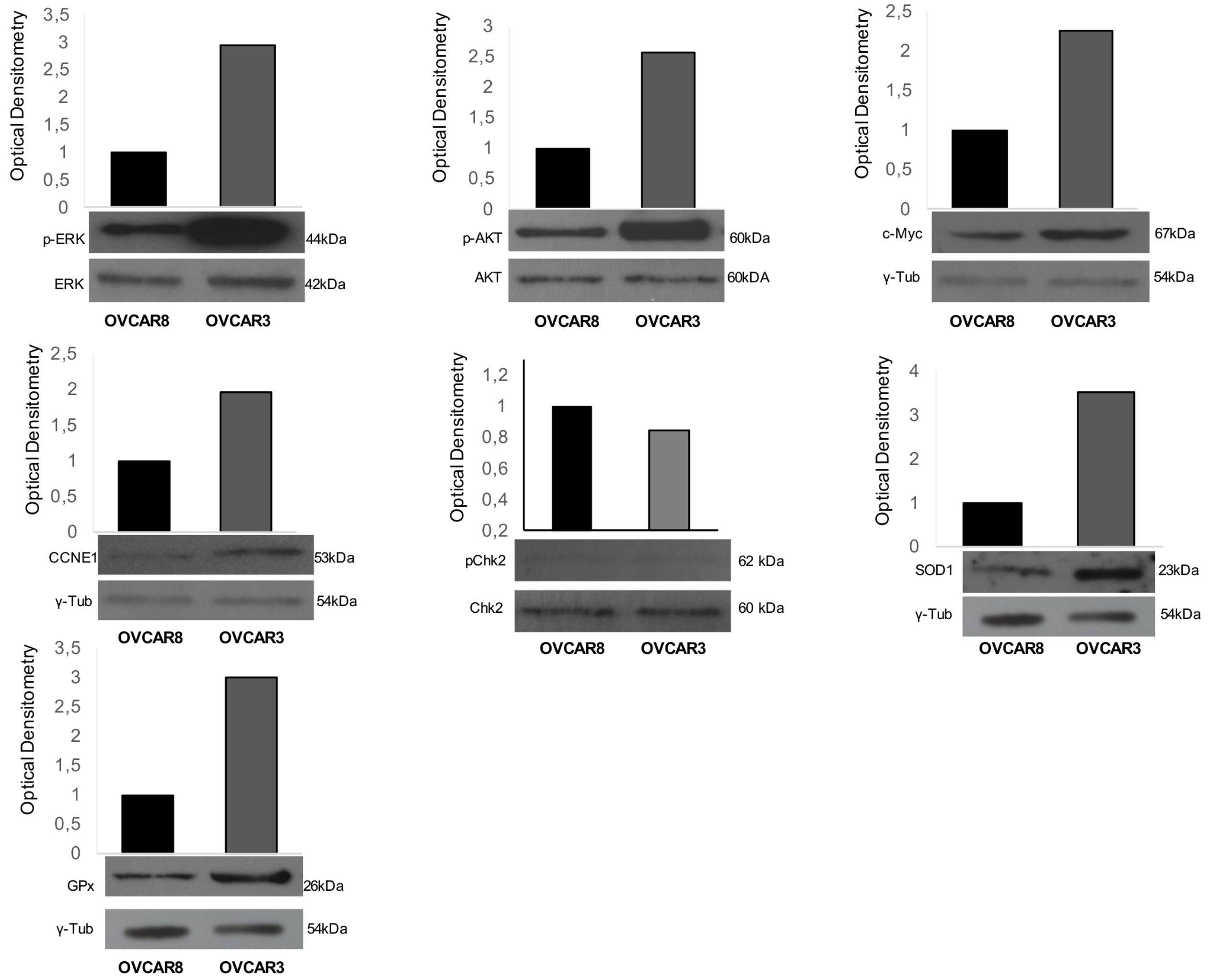

Fig.S1 WB analysis of SOD1, GPx, CCNE1, c-Myc, ERK/pERK, AKT/pAKT and Chk2/pChk2 in OVCAR3 and OVCAR8 cells. Representative western blot of antioxidant proteins SOD1 and GPx, CCNE1, c-Myc together with ERK/AKT/Chk2 phosphorilation in OVCAR3 and OVCAR8 cells. γ-tubulin was used as internal control. Each plot has been quantified by using ImageJ software and optical densitometry is reported. WB analysis was performed three times and results were reproducible.

Figure S2

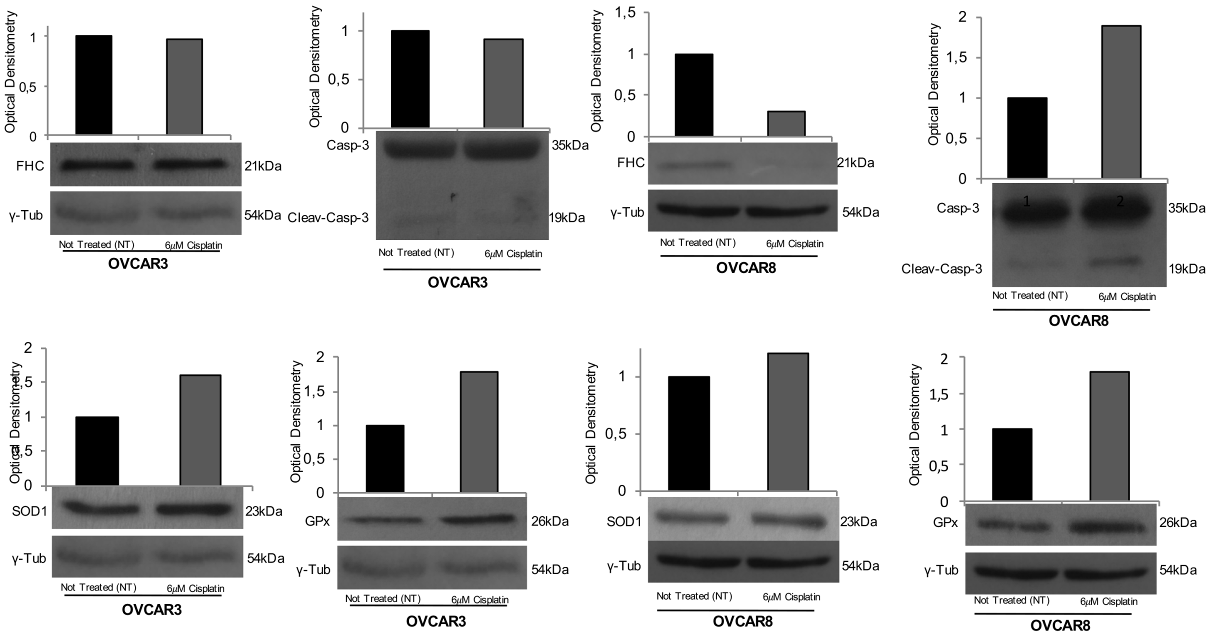

Fig. S2 WB analysis of FHC, Caspase 3/cleaved Caspase 3, SOD1 and GPx in OVCAR3 and OVCAR8 cells untreated or treated with 6µM cisplatin. Representative western blot of FHC, Caspase 3/cleaved Caspase 3, SOD1 and GPx in OVCAR3 and OVCAR8 cells untreated (NT) or treated with 6µM cisplatin. γ-tubulin was used as internal control. Each plot has been quantified by using ImageJ software and optical densitometry is reported. WB analysis was performed three times and results were reproducible.

Figure S3

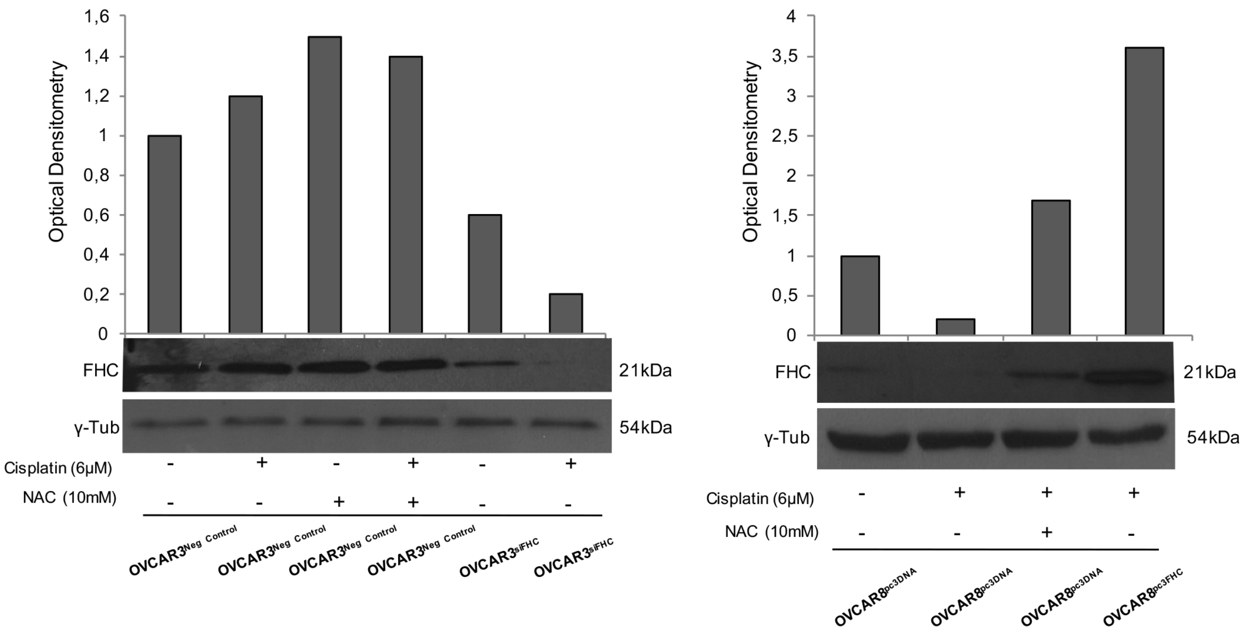

Fig. S3 WB analysis of FHC, Caspase 3/cleaved Caspase 3, SOD1 and GPx in OVCAR3 and OVCAR8 cells upon several treatments. Representative western blot of FHC in OVCAR3$^{Neg\ Control}$, OVCAR3$^{Neg\ Control}$ (6μM) cisplatin, OVCAR3$^{Neg\ Control}$ 10mM NAC, OVCAR3$^{Neg\ Control}$ (6μM) cisplatin/10mM NAC (6μM) cisplatin, OVCAR3^{siFHC}, OVCAR3^{siFHC} (6μM) cisplatin, OVCAR8^{pc3DNA}, OVCAR8^{pc3DNA} (6μM) cisplatin, OVCAR8^{pc3DNA} (6μM) cisplatin/10mM NAC, OVCAR8^{pc3FHC} (6μM) cisplatin. γ-tubulin was used as internal control. Each plot has been quantified by using ImageJ software and optical densitometry is reported. WB analysis was performed three times and results were reproducible.

Figure S4

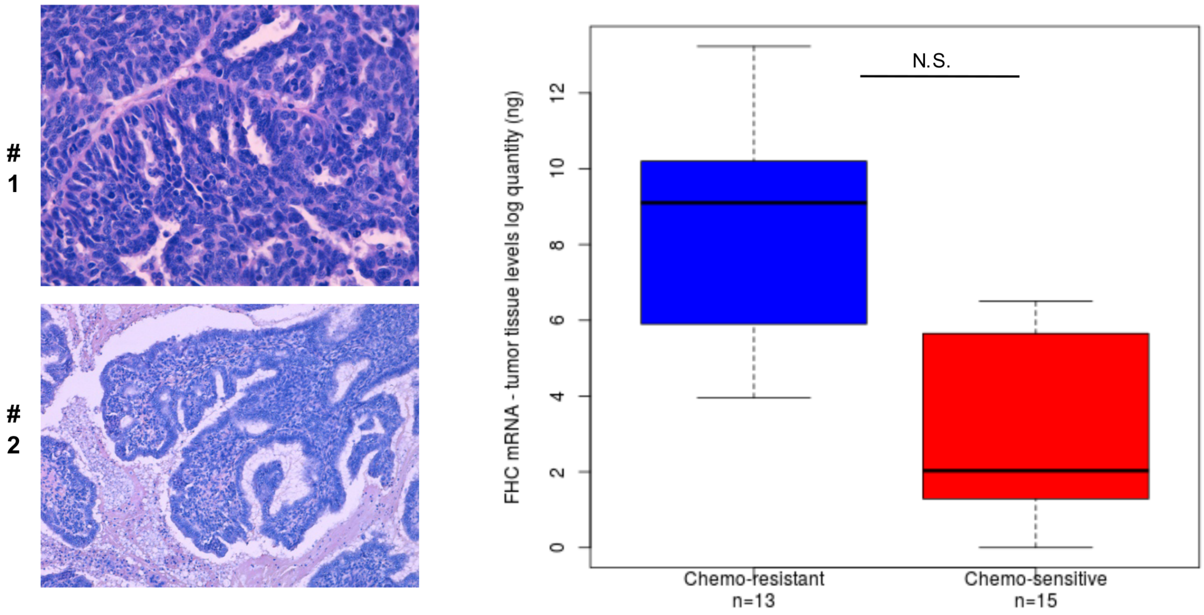

Fig. S4 Analysis of FHC tumor tissue levels in 28 HGSC patients. Representative images of ovarian cancer tissues specimens (left). Box Plot depicting FHC mRNA levels, expressed as log (ng), as assessed by absolute qPCR analysis in cancer tissue specimens of chemo-resistant HGSC patients (n=13) and chemo-sensitive HGSC patients (n=15) (right). Data are not statistically significant (N.S.).
